# A pleasant familiar odor influences perceived stress and peripheral nervous system activity during normal aging

**DOI:** 10.3389/fpsyg.2014.00113

**Published:** 2014-02-17

**Authors:** Pauline Joussain, Catherine Rouby, Moustafa Bensafi

**Affiliations:** Lyon Neuroscience Research Center, CNRS UMR5292, INSERM U1028, University of LyonLyon, France

**Keywords:** olfaction, exposure, stress, mood, physiology, aging

## Abstract

Effects of smells on stress have been demonstrated in animals and humans, suggesting that inhaling certain odorants may counteract the negative effects of stress. Because stress plays a key role in cerebral aging, the present study set out to examine whether positive odor effects on perceived stress can be achieved in elderly individuals. To this end, two groups of aged individuals (*n* = 36 women, aged from 55 to 65 years), were tested. The first group was exposed for 5 days to a pleasant and, by end of exposure, familiar odor (“exposure odor”), whereas the other was exposed to a non-scented control stimulus. Stress and mood states were assessed before and after the 5-day odor exposure period. Psychophysiological markers were also assessed at the end of exposure, in response to the “exposure odor” and to a “new odor.” Results revealed that stress on this second exposure was decreased and zygomatic electromyogram activity was increased specifically in the group previously exposed to the odor (*p* < 0.05). Taken as a whole, these findings offer a new look at the relationship between perceived stress, olfaction and normal aging, opening up new research perspectives on the effect of olfaction on quality of life and well-being in aged individuals.

## INTRODUCTION

In daily life, odors influence behavior and affective states: toxic substances are avoided thanks to the sense of smell, whereas smells are prominent keys to the hedonic pleasure provided by food or perfumes. The relationships between olfaction and affects have been extensively studied in the last decade. This recent research showed that the effects of odors on affective behavior are partly predisposed (Khan et al., 2007; Mandairon et al., 2009; Poncelet et al., 2010b; Joussain et al., 2011; Zarzo, 2011), but are also tuned by learning mechanisms, whether associative learning or mere exposure (Cain and Johnson, 1978; Rouby et al., 2009b; Poncelet et al., 2010a). These affective states influence behavior and mood to the extent that 12 days’ exposure to pleasant odors improved mood in females at midlife, opening new perspectives on the beneficial effect of odor exposure during normal aging (Schiffman et al., 1995). These effects were documented by Schiffman et al. (1995) for specific mood states (tension and depression), and very little is known about odor effects on other individual psychological and physiological responses such as stress. The aim of the present study was to test the effect of odor exposure on perceived stress during normal aging.

Studying the effect of odors on stress during normal aging would be particularly valuable because stress plays a key role in brain aging: reduced resilience in response to changes produced by exposure to a chronic stressor could explain some of the morphological, hormonal and behavioral changes observed in the aged brain (Garrido, 2011). Moreover, focusing on older people is of interest because, despite age-related loss of olfactory function in terms of detection, discrimination, pleasantness and identification (Doty, 1989; Hummel et al., 2007; Joussain et al., 2013), the subjective importance of olfaction remains unchanged (Croy et al., 2010).

Positive effects of odorants on stress were demonstrated in rats and mice and also in young adult humans (Fukada et al., 2007; Oka et al., 2008; Ito et al., 2009; Nikaido and Nakashima, 2009; Mezzacappa et al., 2010), suggesting that inhaling certain odorants may counteract the negative effects of stress. The present study tested the general hypothesis that odor exposure decreases stress in aged individuals. Women around the menopause in particular were chosen because changes around the menopause induce both physiological and social stress, added to aging effects as such [see (Pouliot et al., 2008)]. A second reason for limiting the study to women was that choosing female subjects also allowed the olfactory exposure procedure to be hidden inside an everyday activity that is far more frequent in women: a skin care routine. Finally, because stress is a multidimensional state including psychological and physiological components, both perceived stress and peripheral nervous system activity [heart rate, respiration and facial electromyogram (EMG)] were recorded.

Participants were randomly assigned to either a “test group,” in which the odorized source object consisted of scented cosmetic creams, or a “control group” in which similar but unscented creams were used. Participants were not aware of this difference and no mention was made of the presence of a perfume. They were tested in two separate sessions. In the first session, on arrival in the laboratory they were asked to complete a subjective questionnaire comprising perceived stress and mood items. They were then given the cosmetics, and the procedure to be followed during a week of application was explained to them. After 5-days’ exposure, they came back to the lab for a second session and completed the subjective questionnaire again. Afterward, a within-subject design was used such that each subject (in either group) was tested with the “exposure-odor” (which had been present in the cosmetic cream of the “test group” but not in that of the controls) and a “new-odor” (not present in either of the cosmetics) while physiological parameters were recorded.

Specific hypotheses were that: (i) odor exposure should decrease stress and modulate mood (increase positive mood and decrease negative mood); (ii) odor exposure should reduce the physiological response associated with stressful situations or aversive events (decrease heart rate and respiratory rhythm) and increase physiological response to positive affects [increase zygomatic activity, since a positive correlation between the activity of this facial muscle and sensorial pleasure was observed in past studies (Lang et al., 1993; Sloan et al., 2002)]; and (iii) the odor used in the exposure procedure should become more pleasant and more familiar in the “test group”.

## MATERIALS AND METHODS

### SUBJECTS

Forty-eight women aged between 55 and 65 years participated in the experiment after giving informed consent to procedures that had been approved by the Lyon Committee for the Protection of Human Subjects and conducted in accordance with the Declaration of Helsinki. They were screened for history of neurological disease or injury and of nasal insult. They were randomly assigned to either a “test group” in which the effect of an odorized stimulus (exposure-odor) was evaluated, or a “control group” using the same (but unscented) stimulus. Only 36 of the original 48 subjects (17 from the test group and 19 from the control group) could be analyzed, due to missing questionnaire data and/or problems in recording physiological data. The two groups did not differ in age [mean+/-SEM: test group, 58.6+/-0.9 years; control group, 59.8+/-0.7 years; *F*(1,34) = 1.255, *p* > 0.05]. It is noteworthy that all the women reported menopausal symptoms but none were currently under hormonal replacement therapy. Menopausal age did not differ between groups [mean+/-SEM: test group, 7.3+/-1.3 years; control group, 9.2+/-0.7 years; *F*(1,34) = 1.873, *p* > 0.05].

Because anhedonia may influence hedonic perception of odors (Pouliot et al., 2008), the anhedonia level of each woman was assessed on the Physical Anhedonia Scale (Chapman et al., 1976), a 61-item true/false inventory. Anhedonia is measured from assertions about stimuli and situations which are socially recognized as pleasant. Thus, the anhedonia scale measures disagreement with the positive semantic encoding of sensory experience, or how much subjects distance themselves from positive emotional stimuli. The questionnaire shows significant reliability and has been validated in previous non-olfactory studies (Loas et al., 1996; Dubal et al., 2000). Possible scores range from 0 to 61 (a low score corresponding to a low degree of anhedonia). Anhedonia scores did not differ between the two groups [mean+/-SEM: test group, 13.5+/-1.4; control group, 14.5+/-1.5; *F*(1,34) = 0.251, *p* > 0.05], Finally, subjects’ olfactory performance was estimated on the ETOC (Thomas-Danguin et al., 2003). The ETOC comprises 16 blocks of four flasks. Only one flask per block contains an odorant. For each block, participants are asked firstly to detect which flask contains an odorant and secondly to identify the detected smell. Identification is assessed by a multiple-choice procedure in which participants must select the correct descriptor out of four. Detection scores range from 0 to 16 and are an indicator of sensitivity; identification scores also range from 0 to 16, but only odors that have been correctly detected are taken into account, thus reducing the probability of fortuitous correct identification. Neither detection [mean+/-SEM: test group, 14.9+/-0.4; control group, 14.5+/-0.3; *F*(1,34) = 0.627, *p* > 0.05] nor identification scores [mean+/-SEM: test group, 12.8+/-0.4; control group, 11.9+/-0.4; *F*(1,34) = 1.959, *p* > 0.05] differed between groups.

### PROCEDURE

Participants were tested in two separate sessions. In the first session, on arrival in the laboratory they were asked to complete a subjective questionnaire combining perceived stress assessment and positive and negative mood items. Practically, they were asked to rate what degree of stress they perceived on a single 9-point visual scale from 1 (“not at all stressed”) to 9 (“very strongly stressed”). In addition, they were asked to rate how strongly they were experiencing each of a number of positive (amused, calm, confident, content, happy, interested) and negative emotional states (afraid, angry, annoyed, anxious, bored, contemptuous, disgusted, sad), using the same 9-point scales from 1 (“not at all amused,” etc.) to 9 (“very strongly amused,” etc.). “Sexually aroused” was also added as an item and used as a descriptor. This questionnaire was validated in previous olfactory studies (Bensafi et al., 2003, 2004).

The procedure to be followed during the week of exposure was then detailed. Practically, they were first given two cosmetic creams (one for the face and one for the body). They were explained that the main aim of the study was to assess the impact of these creams on mood and emotion. They were instructed to use the creams each morning for 5 days; they were not allowed to use their normal scented cosmetics during that week and were restricted to non-perfumed toiletries during the course of the study. They were not asked to assess any physical or sensory attributes of the creams. However, they were asked to assess their mood (on the subjective questionnaire used in the first session) every morning before and after application of the cosmetics. Participants who did not fill in all questionnaires during the 5 days were excluded from analysis. In the test group, the cosmetics were odorized with a pleasant floral odor (“exposure odor”: citrus, resinous notes, Symrise^®^), but were non-odorized in the control group. It is noteworthy here that the cover story was exactly the same in both groups: the smell of the cosmetics was never mentioned in any instructions, whichever the group.^®^

After the week of exposure, subjects came back to the lab for a second session and completed the subjective questionnaire again. A within-subject design was then implemented such that each subject (in either group) was tested with the “exposure odor” (that had been present in the test group’s but not the control group’s cosmetics) and another pleasant floral odor (“new odor”: green, woody notes, Firmenich^®^) while physiological parameters were recorded. It is noteworthy that both, the “exposure odor” and the “new odor” were selected because they were a priori pleasant and included olfactory notes used in perfumery (e.g., floral, citrus, resinous, green, woody notes).

All testing was performed in a ventilated room designed specifically for olfactory experiments. The experimenter fitted the subject with the peripheral nervous system recording and odor diffusion equipment. Once peripheral nervous system measurements stabilized, recording was initiated to obtain a psychophysiological baseline. The two odor conditions (“exposure odor” and “new odor”) were presented randomly (i.e., individual order for each subject) via an olfactometer (Rouby et al., 2009a). There was no verbal interaction between investigator and subject during the recording session and participants were asked to relax as much as possible. At the end of the session, they were asked to rate the intensity, familiarity, and pleasantness of both odors on a scale from 1 (not at all intense, pleasant, familiar) to 9 (very intense, pleasant, familiar).

### ODOR DIFFUSION AND OLFACTOMETRY

Pure air was delivered by a compressor and cleaned by an active carbon filter, then carried to the olfactometer input line (6 mm diameter, 5 m length tube). A manometer allowed selection of air input pressure. The air then entered two channels: (1) the air-carrier channel and (2) the odorized channels (one channel per odorant). Each odorized channel contained a glass tube with polypropylene marbles, in which one of the two odorants was adsorbed. Thus, at the exit from each channel, an electric valve could be programmed closed or open in order to determine which odorant would be pushed into the airflow, and for how long. This allowed opening/closure of each valve, and thus stimulus duration (60 s, two presentations of each odor), to be controlled. The interstimulus interval (ISI) was between 60 and 120 s. Odor concentration was 0.5% vol/vol in the cosmetic creams, and a similar perceived intensity was set for the smell diffused from the olfactometer. Carrier airflow was constant, at 1,500 ml/min, and the flow rate of each electric valve was set at 100 ml/min; output odorous air was led through a 4 mm tube (20 cm length) into the nasal mask; both nostrils were stimulated.

The ventilated and refreshed experimental room comprised two spaces: one for the experimenter and one for the subject. The experimenter’s space contained the computer controlling the olfactometer’s physiological parameters; the subject’s space included the output part of the olfactometer and a computer screen and mouse to read instructions and give responses after the session.

### PHYSIOLOGICAL PARAMETERS

In previous studies, olfactory compounds induced psychophysiological responses related to changes in electrodermal response, systolic blood pressure, EMG, respiration, and finger pulse rate (Alaoui-Ismaili et al., 1997a, b; Bensafi et al., 2002; Delplanque et al., 2009; Croy et al., 2013). In the present study, psychophysiological effects were measured on three parameters that were simultaneously and continuously recorded and displayed during the experiment: facial zygomatic EMG, Finger pulse frequency (FPF) and respiratory rate (RR). Electrodermal response magnitude was not used, because it is highly variable in the elderly, some aged subjects showing great variation and others no significant response (Abriat et al., 2007). All parameters were sampled and recorded at 32 Hz. Data were converted and amplified via a 8-channel Procomp+ amplifier (Thought Technology, Montreal, QC, Canada), and displayed, stored, reduced and analyzed off-line.

Facial EMG, expressed in microvolts (μV), was measured using miniature Ag/AgCl electrodes (diameter, 0.8 cm) placed on the zygomatic muscle after cleaning the skin with alcohol. The electrodes were filled with electrode paste and attached with adhesive disks. EMG activity was measured on a PROCOMP+ amplifier (Thought Technology), with band pass filtered from 20 to 1,000 Hz. Data were reduced to EMG area under the curve, calculated during a time window of 10 s after odor diffusion. This time window was chosen to limit analysis to facial mimics induced by the olfactory stimuli.

Changes in abdominal circumference with respiration were measured using a respiratory belt transducer (100 cm rest length, 10 cm maximum elongation, 3.5 cm width), responding linearly to changes in length. Data were reduced to RR, calculated during both 60-s periods of odor diffusion.

Finger pulse frequency was measured using a photoplethysmographic probe (3.2 cm/1.8 cm, LED type photodetector) placed on the thumb of the non-dominant (i.e., left) hand. Data were reduced to pulse rate in beats per minute (BPM).

### DATA ANALYSIS

Stress and mood data were analyzed in two ways. First, they were expressed as differences in rating between sessions 1 and 2 (session 2 minus session 1: “long-term effect” analysis). Second, stress and mood data during the week of application were expressed as differences in rating before and after daily use of cosmetics (after minus before: “application effect” analysis) and averaged across the 5 days. In both analyzes, stress and mood data were compared on one-way ANOVA, with group (“test group” vs. “control group”) as between-subjects factor.

Physiological data compared on ANOVA for each physiological parameter, with condition (“exposure odor” and “new odor”) and time (“first presentation” and “second presentation”) as within-subject factors and group (“test group” vs. “control group”) as between-subjects factor. For physiological data, if significant “group”^*^”condition” or “group”^*^”condition”^*^”time” interactions were observed, the analysis was followed by paired comparisons (without setting corrections for multiple comparison, since the hypotheses were specific).

## RESULTS

### EFFECTS ON STRESS AND MOOD

**FIGURE 1:**
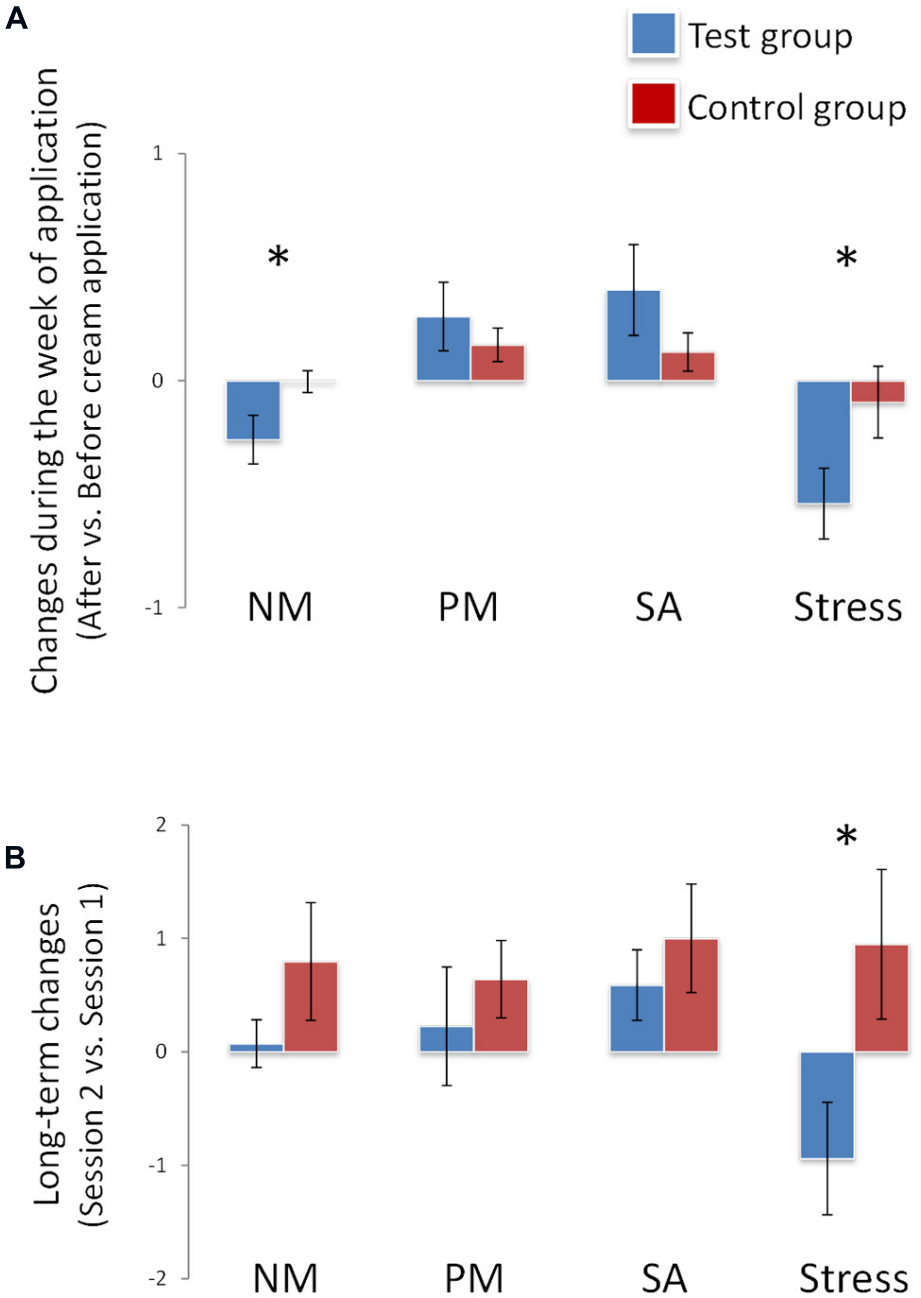
**Effects on mood, sexual arousal, and stress.** (**A**) During the week of application, negative mood and stress decreased significantly (*) in the test group vs. the control group. Stress decreased significantly (*) in the test group vs. the control group at end of the week of application (long-term changes, **B**). NM, PM, SA: respectively, negative mood, positive mood and sexual arousal. *p <0.05. Data are expressed as means and SEM.

During the week of application, a significant effect of group on mood was observed: negative mood decreased in the test group compared to the control group [*F*(1,34) = 5.036, *p* = 0.03]. This effect was accompanied by an effect on stress: perceived stress decreased in the test group compared to the control group [*F*(1,34) = 4.018, *p* = 0.05]. No effect of group was observed for positive mood [*F*(1,34) = 0.584, *p* > 0.05] or sexual arousal [F(1,34) = 1.718, p > 0.05] (**Figure 1A**; **Table 1**).

**Table 1:** Mood, sexual arousal, and stress changes (mean and SEM) during the week of application (after vs. before the daily application of the odorized cosmetics (test group) and non-odorized cosmetics (control group).

	Test group		Control group	
	Mean	SEM	Mean	SEM
Negative mood	-0.26	0.11	0.00	0.05
Positive mood	0.28	0.15	0.16	0.07
Sexual arousal	0.40	0.20	0.13	0.08
Stress	-0.54	0.16	-0.09	0.16

After the week of exposure, the group effect for stress was replicated [*F*(1,34) = 5.040, *p* = 0.03]: the test group felt less stress than the control group. However, no significant differences between groups were observed for sexual arousal [*F*(1,34) = 0.497, *p* > 0.05], negative mood [*F*(1,34) = 1.534, *p* > 0.05] or positive mood [*F*(1,34) = 0.461, *p* > 0.05] (**Figure 1B**; **Table 2**).

**Table 2:** Mood, sexual arousal, and stress changes (mean and SEM) between the second session and the first session in the test group and the control group.

	Test group		Control group	
	Mean	SEM	Mean	SEM
Negative mood	0.07	0.21	0.80	0.52
Positive mood	0.23	0.52	0.64	0.34
Sexual arousal	0.59	0.31	1.00	0.48
Stress	-0.94	0.50	0.95	0.66

### EFFECTS ON PERIPHERAL NERVOUS SYSTEM ACTIVITY

**FIGURE 2:**
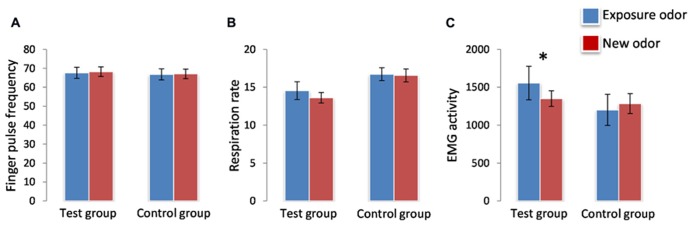
**Effects on Finger Pulse (**A**), Respiration (**B**), and EMG activity (**C**).** Zygomatic EMG increased significantly (*) in response to the exposure odor vs. the new odor in the test group but not in the control group. Finger pulse and respiration activities did not change as a function of odor type (exposure odor vs. new odor) in the test group or the control group. *p <0.05. Data are expressed as means and SEM.

Finger pulse frequency showed a significant effect of time [*F*(1,34) = 5.455, *p* = 0.0256] reflecting a general decrease of FPF from the first presentation (mean+/-SEM: 67.85+/-1.922) to the second presentation (mean+/-SEM: 66.79 +/-1.81). However, no significant effects of group [*F*(1,34) = 0.089, *p* > 0.05] and odor [*F*(1,34) = 1.161, *p* > 0.05] and no significant odor^*^group or odor^*^group^*^time interactions were observed (**Figure 2B**). RR showed no significant effect of group [*F*(1,34) = 4.021, *p* > 0.05], odor [*F*(1,34) = 1.321, *p* > 0.05] and time [*F*(1,34) = 0.322, *p* > 0.05], and no significant odor^*^group or odor^*^group^*^time interaction (**Figures 2A,B**; **Table 3**).

**Table 3:** Physiological responses to the exposure odor and the new odor (mean and SEM of EMG area under the curve, Respiratory rate or RR and Finger pulse frequency or FPF) in the test group and the control group.

		Test group		Control group	
		Mean	SEM	Mean	SEM
EMG	“Exposure odor”	1555.31	220.43	1200.19	103.11
	“New odor”	1350.77	204.93	1284.55	131.01
RR	“Exposure odor”	14.56	1.17	16.72	0.69c
	“New odor”	13.62	0.85	16.55	0.86
FPR	“Exposure odor”	67.62	2.92	66.78	2.54
	“New odor”	68.21	2.89	67.05	2.47

For EMG, however, a significant odor*group interaction [*F*(1,34) = 16.555, *p* = 0.0003], but no significant group [*F*(1,34) = 0.823, *p* > 0.05], odor [*F*(1,34) = 2.744, *p* > 0.05] or time [*F*(1,34) = 0.757, *p* > 0.05] effect, was observed: EMG activity was greater for the exposure odor than for the new odor in the test group (*p* = 0.003) but not in the control group (*p* > 0.05; **Figure 2C**; **Table 3**). It is noteworthy here that these EMG effects were accompanied by perceptual differences in each group: (i) in the test group, the exposure odor was perceived as more pleasant (*p* = 0.042), more familiar (*p* = 0.041) but not more intense (*p* > 0.05) than the new odor, (ii) and as more familiar (*p* = 0.010), but not more pleasant (*p* > 0.05) and more intense (*p* > 0.05) in the control group (**Table 4**).

**Table 4:** Intensity, pleasantness, and familiarity ratings (mean and SEM) of the exposure odor and the new odor in the test group and the control group.

		Test group		Control group	
		Mean	SEM	Mean	SEM
Intensity	“Exposure odor”	5.94	0.42	6.84	0.38
	“New odor”	4.88	0.51	6.32	0.50
Pleasantness	“Exposure odor”	6.53	0.34	6.58	0.33
	“New odor”	5.41	0.43	6.05	0.44
Familiarity	“Exposure odor”	6.35	0.49	7.03	0.30
	“New odor”	4.79	0.59	5.47	0.61

## DISCUSSION

The present study tested the hypothesis that regular exposure to an odor in a natural setting decreases stress and modulates peripheral nervous system response in aged women. Daily olfactory exposure did indeed modify perceived stress: compared to controls, test group subjects showed decreased negative mood and stress during the week of regular exposure. Although the effect on mood was not confirmed one week later, the stress effect persisted at the second session: the test group showed less stress than the control group after the week of exposure. These findings are in line with animal and human studies showing an influence of odors on stress: for example, “green odors” have been shown to exert anxiolytic and stress-reducing effects in human subjects (Oka et al., 2008) and also to alleviate stress-induced cardiovascular, hormonal, and behavioral responses in rats (Ito et al., 2009; Nikaido and Nakashima, 2009). Similar effects were recently reported for coconut (Mezzacappa et al., 2010) and rose odors (Fukada et al., 2007). Because stress plays a key role in brain aging, not only exercise but also environmental stimulation can contribute to protecting the aging brain against stressors (Garrido, 2011). In line with this, animal and human studies (van Praag et al., 2000; Mahncke et al., 2006) suggest that there is significant benefit in repeatedly exposing human subjects to sensory cues. The present study extended these findings to olfaction on the one hand and perceived stress on the other.

Interestingly, the observed modulation of stress was accompanied by modified psychophysiological patterns: following stimulation with the test odor (unlike the control odor), zygomatic EMG activity increased in the test group but not in the control group. This effect on facial EMG activity was associated with a modulation of odor hedonic response: the exposure odor was perceived as more pleasant than the new odor in the test group but not in the control group, in agreement with the literature on exposure effects in the visual domain (Monahan et al., 2000). It is noteworthy that our study was conducted in women, with results in line with data showing that, in terms of emotional response to odors, women report more frequent evocations of emotional memories by odors and stronger feelings of happiness, sadness and well-being, and reduced stress as a consequence of smelling odors (Martin et al., 2001).

Another result of interest was the greater familiarity of the exposure odor compared to the control odor in both groups. Although it was expected that the exposure odor would be rated as more familiar than the control odor in the test group, this was not assumed for the control group. This reflects the fact that, overall, the exposure odor was perceived as more familiar, raising the question as to whether the present effect on stress and physiology could be obtained with an unfamiliar odor. This greater familiarity does not, however, weaken the strength of our finding, but leads us to consider the exposure odor as being a familiar odor. In sum, the present study suggests that stress (and at least negative mood during the week of exposure) and physiology in elderly people can be influenced by repeated exposure to a pleasant and familiar odor in a natural setting.

That a pleasant familiar odor influenced stress and physiological response in aged women is a novel finding. The question arises as to the route by which this effect is produced. One possibility would involve a direct effect on neural activity in the substrates of mood and stress, but mediated by the olfactory system. Such a path may reflect a privileged relationship between the neural substrates of olfaction and regions of the brain involved in affective processing (Gottfried et al., 2002; Anderson et al., 2003; Rolls et al., 2003; Bensafi et al., 2012).

The present study thus provides the first evidence for an influence of exposure to a familiar odor on perceived stress and facial electromyographic activity in aged women in a natural setting. It is important to mention here that other sensory influences may have accompanied the effect of odor exposure. Indeed, an associative learning linking the exposure odor with a supposedly pleasant touch (tactile stimulation during application of body and facial cosmetics) may have occurred. This possibility is not unlikely since our ecological situation was multimodal, involving olfactory but also visual and tactile stimuli. In such natural settings, it is not easy to isolate the specific influence of touch and smell on stress, and physiology. However, our data shows that the same situation without smell (e.g., control group), did not impact stress and physiology, reflecting that the smell used was a prominent driver of the observed effect.

Besides the above, another question that may be raised concerns potential inter-group differences in individual factors such as age, impaired sensory pleasure or hormonal status. Olfactory function is known to be impaired with age (Doty, 1989; Hummel et al., 2007), and odor hedonic perception was also found to be modified in aged people (Joussain et al., 2013). However, this possibility is ruled out in the present case by the fact that the two groups of aged women did not differ in mean chronological age or anhedonia level. Moreover, although we cannot confirm that the hormonal status of the women in the two groups was equivalent, as we did not measure it, no women in either group were taking hormonal replacement therapy and there was no difference in mean menopausal age.

In conclusion, notwithstanding the above reserves, the present study offers new insight into the effect of exposure to a familiar pleasant odor on perceived stress and physiology. The effects observed here cannot be explained adequately by age, menopausal age or differential impairment of sensory pleasure. The present study demonstrates for the first time that a 1-week odor exposure procedure in an ecological setting can modulate stress, and opens up new research perspectives on the effect of olfaction on quality of life and well-being.

## Conflict of Interest Statement

The authors declare that the research was conducted in the absence of any commercial or financial relationships that could be construed as a potential conflict of interest.
